# Association between anxious-depression and cognitive function in community-dwelling older adults: a latent class analysis

**DOI:** 10.3389/fpubh.2026.1851644

**Published:** 2026-06-26

**Authors:** Yiran Deng, Siwen Lv, Letian Yang, Xinyu Gao, Hao Geng, Siwen Liu, Xiaoming Kong

**Affiliations:** 1 School of Mental Health and Psychological Sciences, Anhui Medical University, Hefei, China; 2 Hefei Fourth People’s Hospital, Affiliated Psychological Hospital of Anhui Medical University, Anhui Medical University, Hefei, China

**Keywords:** anxious-depression, cognitive function, latent class analysis, older adults, symptom patterns

## Abstract

**Background:**

Anxiety and depression commonly coexist in later life, yet little is known about the heterogeneous manifestations of anxious–depression symptoms and their cognitive correlates among community-dwelling older adults in China. The purpose of this study is to identify heterogeneous anxious-depression symptom profiles and examine their associations with cognitive function among community-dwelling older adults in China.

**Methods:**

A total of 5,116 community-dwelling older adults aged ≥60 years were surveyed. Anxiety and depressive symptoms were assessed using the 7-item Generalized Anxiety Disorder Scale and the 15-item Geriatric Depression Scale, with latent class analysis (LCA) used to identify anxious–depression symptom patterns. Cognitive function was assessed using the brief Community Screening Inventory for Dementia. Multivariable linear regression analyses were performed to examine the associations between anxious–depression phenotypes and cognitive function.

**Results:**

LCA identified four anxious-depression symptom profiles: high anxious-depression (7.41%), moderate anxious-depression (8.64%), high depression (13.94%), and low anxious-depression (70.01%). After adjusting for covariates, both anxious-depression and depression-dominant phenotypes were significantly linked to lower cognitive function scores (all *p* < 0.001). Individuals with comorbid anxiety and depression showed greater cognitive decline compared to those with predominantly depressive symptoms, with the high anxious-depression group exhibiting the most pronounced reduction in cognitive scores (*p* < 0.001).

**Conclusion:**

Distinct anxious-depression symptom subtypes were identified among community-dwelling older adults. The more pronounced the anxiety and depression symptoms, the poorer the cognitive function. These findings underscore the importance of addressing anxiety and depression in community-based cognitive screening and prevention programs.

## 1 Introduction

Anxiety and depression are the two most common mental health issues among older adults. They have a profound impact on the overall health of the older population (1). Epidemiological surveys conducted across different regions in China have demonstrated marked geographic heterogeneity in the prevalence of these conditions. For instance, the prevalence rates of anxiety and depression among community-dwelling older adults in China Guangxi Zhuang Autonomous Region were 11.42 and 15.94%, respectively (2). In contrast, corresponding prevalence estimates in Shenzhen communities were 11.3% for anxiety and 10.3% for depression (3). These differences may be related to factors such as regional economic development levels, accessibility to healthcare resources, and demographic characteristics. Moreover, the burden of anxiety and depressive symptoms tends to increase with advancing age.

Anxiety and depressive symptoms frequently co-occur in older adults, a phenomenon commonly described as “anxiety–depression comorbidity” or “anxious depression” (4). Epidemiological evidence suggests that approximately 20 to 50% of older adults experiencing depressive symptoms may also exhibit anxiety symptoms (5). This comorbidity not only increases the psychological burden on the older adults but may also accelerate their cognitive decline. Emerging evidence further indicates that the coexistence of anxiety and depression may give rise to more complex health consequences, underscoring the need to better understand its implications for cognitive health in later life.

Both anxiety and depression have been consistently linked to poorer cognitive function (6) and an increased risk of cognitive impairment (7). Research conducted by Ahmed M. Kassem and colleagues (8) has highlighted that anxiety is particularly associated with executive function decline. Previous studies have further demonstrated that anxiety and depressive symptoms may impair multiple cognitive domains, including attention, memory, executive function, and processing speed (9). Anxiety symptoms are often linked to attentional bias and reduced processing efficiency, whereas depressive symptoms are more commonly associated with impairments in episodic memory, psychomotor speed, and executive functioning (10, 11). Since anxiety and depression affect partially overlapping but also distinct cognitive domains, their coexistence may contribute to broader and more severe cognitive dysfunction in older adults. More importantly, the co-occurrence of anxiety and depression may accelerate cognitive decline. A study from Biringer et al. (12) found that compared to isolated anxiety or depressive symptoms, the co-occurrence of anxiety and depressive symptoms is associated with poorer cognitive prognosis.

Although anxiety and depression often coexist, research has suggested that they were two distinct disorders, each possessing its own unique characteristics (13, 14). According to the tripartite model (15, 16), while both anxiety and depression involve general distress or negative emotions (such as restlessness, insomnia, agitation, and irritability), depression is specifically characterized by a lack of positive emotions or anhedonia. In contrast, anxiety is primarily associated with excessive physiological arousal or somatic tension. These emotional and physiological features may contribute to cognitive decline through distinct neurobiological pathways. Anxiety-related physiological hyperarousal has been linked to prolonged hypothalamic–pituitary–adrenal (HPA) axis activation and elevated cortisol levels, which may contribute to hippocampal dysfunction and memory impairment (17). In contrast, depressive symptoms characterized by persistent negative affect and anhedonia may also be associated with dysfunction in fronto-limbic brain networks, reduced dopaminergic activity, and decreased cognitive processing efficiency (18). Therefore, the coexistence of anxiety and depressive symptoms may exert additive or synergistic effects on cognitive functioning through multiple overlapping neurobiological mechanisms. Recent studies (5, 19) have employed latent class analysis (LCA) to explore patterns of anxiety and depression symptoms and identify their underlying subtypes. As a person-centered statistical approach, LCA enables the identification of unobserved population subgroups based on multivariate response patterns (20). In contrast to traditional scale-based or cutoff-based methods, LCA avoids predefined categories and estimates subgroup membership probabilistically, allowing for a more nuanced characterization of symptom phenotypes. This approach therefore provides a clearer understanding of the co-occurrence of anxiety and depression in community-dwelling older adults (16).

Studies on anxiety and depression among community-dwelling older adults in China have mostly been based on total scores or fixed cut-off thresholds, which may overlook the heterogeneity of symptom dimensions within these disorders. In contrast, our study adopts a person-centered latent class analysis approach, which, using probabilistic models, uncovers distinct patterns of symptom combinations. This method provides a better understanding of the different presentations of anxiety and depression in older adults. To date, large-sample LCA studies in China remain scarce, particularly those investigating the heterogeneous subtypes of anxiety-depression comorbidity and their association with cognitive function in older adults. Previous studies have rarely examined this issue. To address this gap, we analyzed data from 5,116 older adults across seven communities in Hefei. This study provides a theoretical basis for developing precise intervention strategies targeting potential cognitive impairment among older residents in Hefei communities, and also provides new insights into how emotional disorders affect cognitive health in the older adults.

## 2 Methods

### 2.1 Participants

This study conducted a community-based screening survey on the emotional state and cognitive function of older adults from seven communities within Hefei City between June 2024 and January 2025. All eligible older adults within these communities were invited to participate.

In this study, the inclusion and exclusion criteria for participants were: (a) Age 60 years or older; (b) Residing in the community for over 1 year; (c) sufficient hearing, vision, communication ability, and physical condition to complete the interview; and (d) provision of written informed consent. The ability to complete the survey was assessed by trained interviewers during the home visit based on whether participants could understand the questions, communicate their responses, and complete the assessment. Older adults with severe sensory impairment that prevented communication, acute confusion or delirium, marked psychotic symptoms, or severe physical illness precluding participation were not enrolled. Participants were also excluded if they or their families refused consent after explanation, or if they could not be contacted after at least three home visits. Recruitment was conducted through home visits in seven communities in Hefei and therefore represents a community-based non-probability sample. Data collection was performed by well-trained staff from community service centers, and completion of all questionnaires required approximately 20 min.

### 2.2 Variables and tools used

For this survey, we created a research tool using the WeChat mini-program, Wanxin Tong 1.0. This platform was used to collect data on participants’ demographic information, including age, sex, living status, education level, economic income status (yearly income), and social status.

The brief Community Screening Instrument for Dementia (CSI-D) consists of two components: a cognitive test and an informant interview. The composite total score (range: −6 to 9) is calculated by subtracting the informant rating (range: 0–6) from the cognitive score (range: 0–9). The cutoff value distinguishing possible cognitive impairment from normal cognition is 4, with higher scores indicating better cognitive function (21). In this study, the informants were primarily spouses, adult children, or other individuals familiar with the participants’ daily functioning and cognitive status, consistent with the original design of the CSI-D (22). The CSI-D was selected because it has been widely validated for community-based cognitive screening across diverse cultural and educational backgrounds and is considered suitable for large-scale epidemiological studies of older adults (21). However, it should be interpreted as a global screening measure rather than a domain-specific neuropsychological assessment.

Depressive symptoms were assessed using the validated Chinese version of the 15-item Geriatric Depression Scale (GDS-15), which comprises 15 items in total. Previous studies have demonstrated good reliability and validity of the Chinese GDS-15 among older Chinese adults (23). A total score >4 was used to indicate depressive symptoms (24). The 7-item Generalized Anxiety Disorder Scale (GAD-7) was used to assess anxiety symptoms in older adults. Previous studies have demonstrated good psychometric properties of the Chinese GAD-7 in Chinese older adults (25). A total GAD-7 score ≥5 was considered indicative of anxiety symptoms (26).

### 2.3 Statistical analysis

Mplus (version 8.3, Muthén and Muthén) was used to perform latent class analysis (LCA) of anxiety and depression symptoms among community-dwelling older adults. Based on the similarity displayed in subjects’ response patterns, LCA categorizes them into more homogeneous groups, each with their own symptom endorsement probability. We conducted LCA on all items of the GAD-7 and GDS-15, dichotomizing each item (i.e., no symptoms = 0, symptoms present = 1). Specifically, the GAD-7 response option “Not at all” was recoded as “No symptoms,” while the options “A few days,” “Half the days,” and “Almost every day” were recoded as “Symptoms present.” The GDS-15 response options remained unchanged. We selected information criteria (ICs) including Bayesian information criteria (BIC), Akaike information criteria (AIC), and sample-size adjusted BIC (a-BIC) in which lower values suggest better model fit. It has been reported in a study (27) that BIC performed better than other ICs. We also used the Vuong-Lo–Mendell–Rubin likelihood ratio test (LMR-LRT) and boot-strapped likelihood ratio test (BLRT) to evaluate whether a k-class model fits better than a k-1 class model. An entropy value closer to 1 indicates better classification accuracy. In selecting the final model, the number of subjects in each class was also considered for parsimony reasons.

The probability levels for the optimal model are defined as follows: Low probability: ≤0.15 Medium probability: 0.16–0.59 High probability: ≥0.6 (28). After determining the optimal model, the remaining statistical analyses were conducted by SPSS (version 27, IBM corporation). Continuous variables were described using mean (standard deviation, SD), while categorical variables were described using frequency (percentage). Intergroup comparisons were performed using chi-square tests and analysis of variance (ANOVA). For each model we reported the estimates beta coefficient, survey adjusted standard error for correct inferences, and two tailed *t*-test *p*-values for statistically significant associations (i.e., *p* < 0.05). *Post hoc* comparisons between groups were performed using the Bonferroni multiple comparison correction method.

### 2.4 Ethics approval

All experiments were conducted in accordance with the Declaration of Helsinki and institutional guidelines, and were approved by the Clinical Research Ethics Review Committee of Hefei Fourth People’s Hospital [HFSY-IRB-YJ-KYXM-KXM (2024–100-001)]. Experimental protocols were committee-approved, informed consent was obtained from participants/guardians, and personal identifiers were omitted.

## 3 Results

### 3.1 Sample characteristics

A total of 15,696 community-dwelling older adults were initially approached for participation in this study. Of these, 3,679 individuals refused to participate in the survey and 2,482 could not be reached after three contact attempts, resulting in 39.3% of participants being excluded prior to assessment. Consequently, 9,535 participants completed the survey and were considered eligible for further assessment.

**Figure 1:**
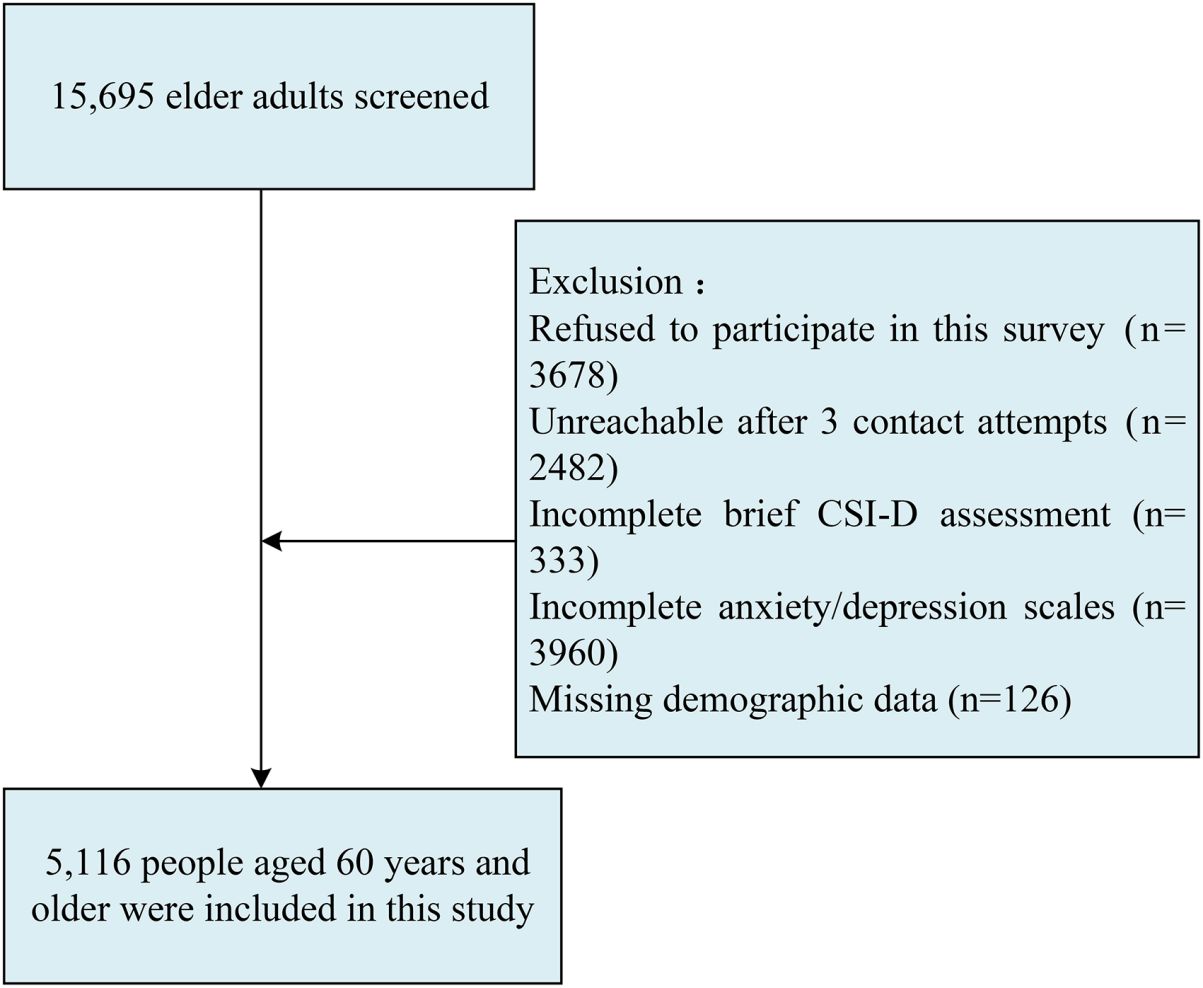
The sampling framework of this study. A total of 15,696 community-dwelling older adults were initially approached for participation in this study. Participants were excluded prior to assessment because they refused to participate in the survey (*n* = 3,679) or could not be reached after three contact attempts (*n* = 2,482). Among the eligible participants, exclusions from the final analysis were due to incomplete anxiety and depression scale data (GAD-7 or GDS-15; *n* = 3,960), incomplete brief CSI-D assessments (*n* = 333), or missing demographic information (*n* = 126). Ultimately, 5,116 community-dwelling older adults with complete emotional and cognitive assessment data were included in the final analysis.

Among these participants, 3,960 had incomplete anxiety and depression scale data (GAD-7 or GDS-15), 333 had incomplete brief CSI-D assessments, and 126 had missing demographic information. Therefore, 46.3% of eligible participants were excluded from the final analysis because of incomplete demographic or core scale data. Ultimately, 5,116 community-dwelling older adults with complete emotional and cognitive assessment data were included in the final analysis (Figure 1).

Comparisons between included and excluded participants are presented in Supplementary Table S1. Excluded participants were significantly older, more likely to be female, and had lower educational levels than included participants (all *p* < 0.05).

In total, 5,116 participants were included in the final analysis (mean age = 69.5 ± 7.3 years; range: 60–99), and 49.3% were male. Table 1 showed that 8.0% live alone, 54.3% had an annual income ≥30,000 RMB, and 45.9% had at least a high school education. Most (83.9%) engaged in social activities. Anxiety and depression were present in 4.4 and 10.6% of participants, respectively, while the cognitive impairment rate was 5.1%, as detected by the CSI-D scale.

**Table 1:** Basic information of community seniors.

Variables	Groups	*N*	M(SD)/%
Age		5,116	69.5(7.341)
Sex			
	Male	2,524	49.3
	Female	2,592	50.7
Living status			
	With household member(s)	4,708	92
	Living alone	408	8
Yearly income			
	≤30,000 RMB	2,339	45.7
	>30,000 RMB	2,777	54.3
Educational level			
	No formal education or primary education	2,769	54.1
	Secondary education	2,347	45.9
Social status			
	Social decline	822	16.1
	Active social interaction	4,294	83.9
Depressive symptoms			
	No	4,572	10.6
	Yes	544	89.4
Anxiety symptoms			
	No	4,891	95.6
	Yes	225	4.4
Possible dementia			
	No	4,854	94.9
	Yes	262	5.1

### 3.2 Anxious-depression phenotypes in LCA analysis

**Figure 2:**
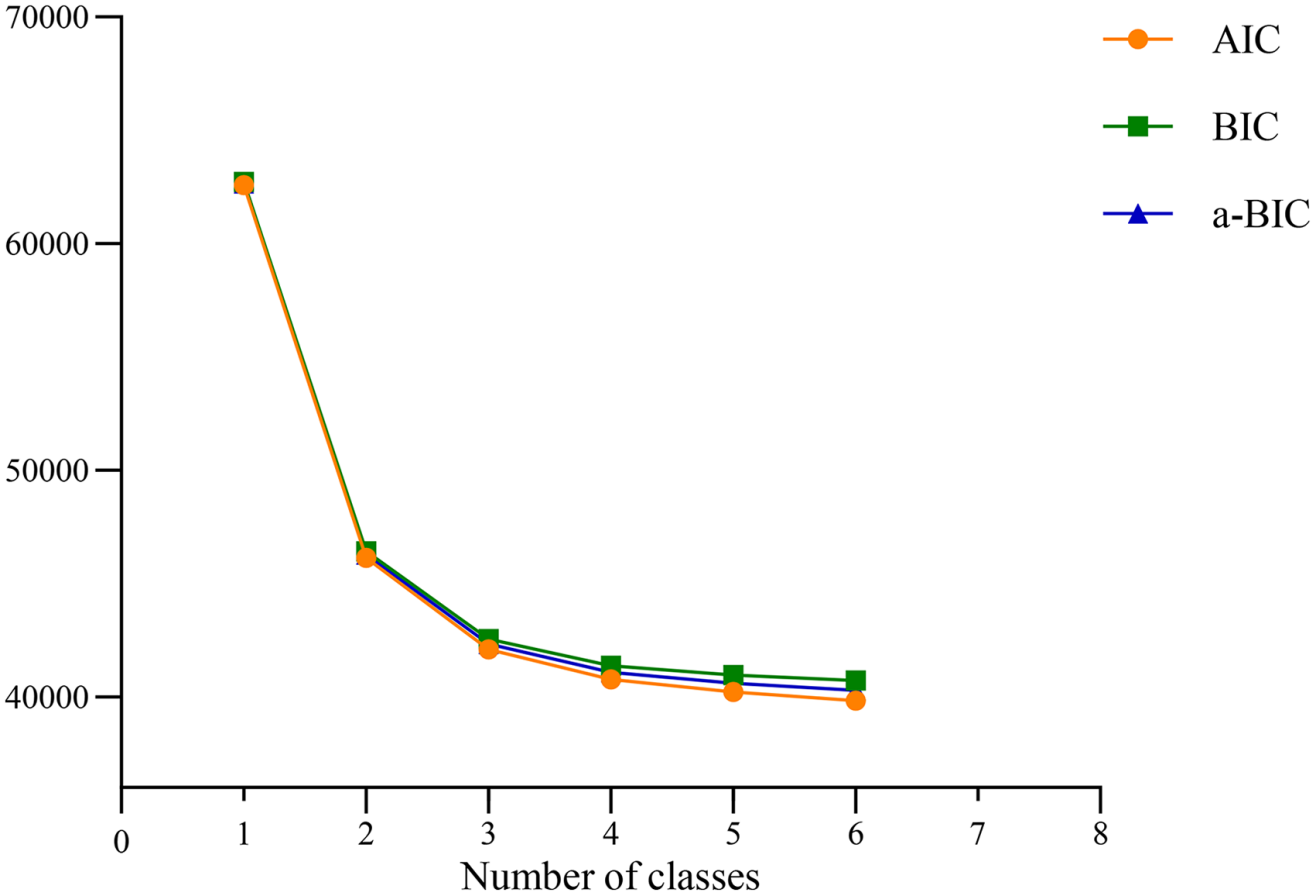
Model fit statistics for derived latent class solutions. This figure corresponds to Table 2 and displays a line chart plotting the values of the three primary information criteria (AIC, BIC, a-BIC) for the 6 classes.

Table 2 and Figure 2 show the fitting information for models 1–6 generated by LCA. Overall, the four-class model was selected as the optimal fit model based on its satisfactory entropy, lower AIC, BIC, and a-BIC values, significant LMR-LRT and BLRT results, and interpretability. Considering the non-significant results of LMR-LRT and BLRT and the principle of parsimony, we excluded the 5- and six-category models. Furthermore, although the entropy of the two-category model (0.957) was the most satisfactory, its BIC value was relatively high and thus it was excluded.

**Table 2:** Fitting statistics for latent class models from 1 to 6 classes.

Number of classes	LL	AIC	BIC	a-BIC	Entropy	BLRT	LMR-LRT	Proportion of each category in the models (%)					
								Class1	Class2	Class3	Class4	Class5	Class6
1	−31268.1	62580.29	62724.17	62654.26									
2	−23018.4	46126.7	46421.01	46278.01	0.957	<0.001	<0.001	14.249	85.751				
3	−20984.9	42105.79	42550.51	42334.43	0.934	<0.001	<0.001	10.256	19.535	70.209			
4	−20294.6	40771.18	41366.33	41077.17	0.941	<0.001	<0.001	7.408	8.64	13.937	70.016		
5	−19991.1	40210.17	40955.74	40593.49	0.944	0.2013	0.1992	8.366	4.066	9.93	70.465	7.174	
6	−19,776	39826.09	40722.09	40286.75	0.942	0.0515	0.0316	6.724	6.372	1.192	8.346	7.819	69.547

AIC, Akaike information criteria; BIC, Bayesian information criteria; a-BIC, Sample-size adjusted Bayesian information criteria; LMR-LRT, Vuong-Lo–Mendell–Rubin likelihood ratio test; BLRT, Bootstrap likelihood ratio test.

**Figure 3:**
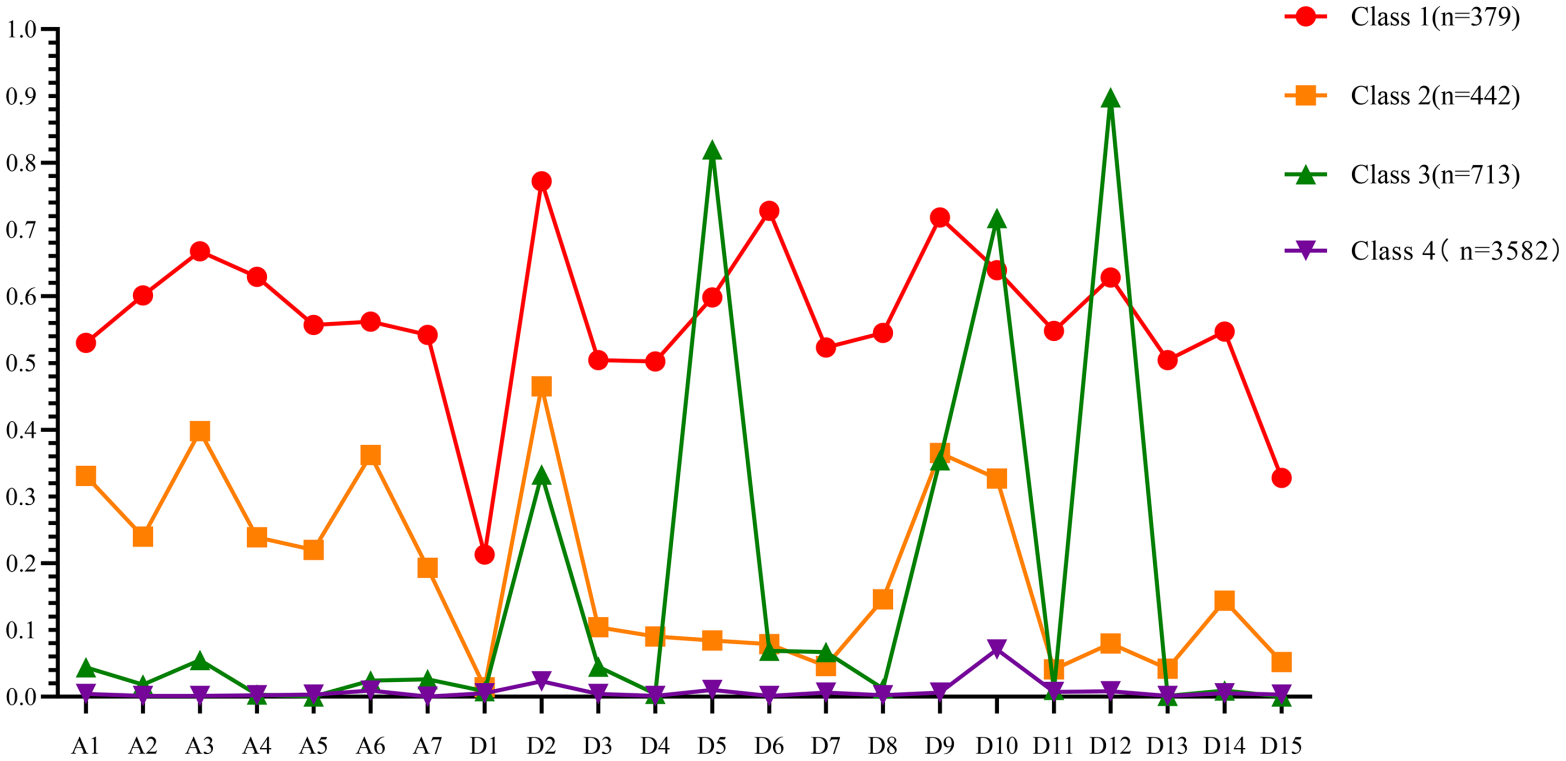
Symptoms profiles in the 4-class model based from the GDS-15 and GAD-7. Probability graphs of symptoms across the four model categories. This figure shows the probability of each item in the GDA-15 and GAD-7 within each potential group. Y-axis: Probabilities. X-axis: A1. Feeling nervous, anxious or on edge; A2. Not being able to stop or control worrying; A3. Worrying too much about different things; A4. Trouble relaxing; A5. Being so restless that it is hard to sit still; A6. Becoming easily annoyed or irritable; A7. Feeling afraid as if something awful might happen; D1. Satisfied*; D2. Dropped activities and interests; D3. Empty; D4. Bored; D5. Good spirits*; D6. Afraid something bad is going to happen; D7. Happy*; D8. Helpless; D9. Prefer to stay at home; D10. Subjective memory complaints; D11. Wonderful to be alive*; D12. Full of energy; D13. Helpless: D14. Think most people are better off: D15. Worthless (*: Reverse coding).

The probabilities of symptom endorsement for each class of the optimal model are presented in Figure 3 and Table 3. The first class (*n* = 379, 7.41% of participants) was characterized by moderate to high probabilities (0.15–0.90) for most anxiety and depression symptoms, and was thus labeled as the high anxious-depression group (High co-occurrence of anxiety and depression symptoms). The second class (*n* = 442, 8.64% of participants) was characterized by anxiety and depression symptom item probabilities at 0.2–0.5, overall lower than the first group. Consequently, this group was labeled as the moderate anxious-depression group. In the third class (*n* = 713, accounting for 13.94% of participants), the probabilities for some depressive items (D5, D10, and D12) were extremely high, even exceeding those of the first class, reaching 0.82, 0.717, and 0.898 respectively; however, the overall probability for anxiety items remained below 0.15. Therefore, this group was labeled as the high depression (where depressive symptoms predominate). In the fourth class (*n* = 3,582, 70.01% of participants), the conditional probabilities for all depression and anxiety items were below 0.1. Therefore, this group was labeled as low anxious-depression.

**Table 3:** Probability of responding to symptom items in the 4-class model based on the GDS-15 and GAD-7.

Scale item	Class 1	Class 2	Class 3	Class 4
A1	0.530	0.331	0.044	0.004
A2	0.601	0.240	0.018	0.001
A3	0.667	0.398	0.055	0.001
A4	0.629	0.239	0.003	0.002
A5	0.557	0.220	0.000	0.003
A6	0.562	0.362	0.024	0.009
A7	0.542	0.193	0.026	0.000
D1	0.213	0.014	0.008	0.005
D2	0.772	0.465	0.333	0.023
D3	0.504	0.104	0.045	0.004
D4	0.502	0.090	0.004	0.001
D5	0.598	0.084	0.820	0.010
D6	0.728	0.079	0.069	0.001
D7	0.523	0.046	0.067	0.006
D8	0.545	0.146	0.012	0.002
D9	0.718	0.365	0.354	0.006
D10	0.639	0.327	0.717	0.071
D11	0.548	0.041	0.010	0.007
D12	0.628	0.080	0.898	0.008
D13	0.504	0.042	0.001	0.001
D14	0.547	0.144	0.009	0.005
D15	0.328	0.052	0.000	0.003

Probability graphs of symptoms across the four model categories. This figure shows the probability of each item in the GDA-15 and GAD-7 within each potential group. Y-axis: Probabilities. X-axis: A1. Feeling nervous, anxious or on edge; A2. Not being able to stop or control worrying; A3. Worrying too much about different things; A4. Trouble relaxing; A5. Being so restless that it is hard to sit still; A6. Becoming easily annoyed or irritable; A7. Feeling afraid as if something awful might happen; D1. Satisfied*; D2. Dropped activities and interests; D3. Empty; D4. Bored; D5. Good spirits*; D6. Afraid something bad is going to happen; D7. Happy*; D8. Helpless; D9. Prefer to stay at home; D10. Subjective memory complaints; D11. Wonderful to be alive*; D12. Full of energy; D13. Helpless: D14. Think most people are better off: D15. Worthless (*: Reverse coding).

The demographic characteristics, anxiety and depression scale scores, and cognitive function scores of the four groups were analyzed (Table 4). Chi-square tests and analysis of variance (ANOVA) revealed statistically significant differences among the four groups in age (*p* < 0.001), living status (*p* < 0.001), educational level (*p* = 0.001), and social status (*p* = 0.004). Consistent with class group characteristics: Group 1 had higher mean GAD-7 and GDS-15 scores than Group 2. Group 3 had higher mean depression scale scores than Group 2, but significantly lower mean anxiety scale scores than Groups 1 and 2, and marginally higher than Group 4. Group 4 had the lowest mean anxiety and depression scale scores.

**Table 4:** Demographic, psychological, and cognitive characteristics of individuals with anxiety and depression symptoms.

Variables	Class 1: high anxious-depression (*n* = 379)	Class 2: moderate anxious-depression (*n* = 442)	Class 3: high depression (*n* = 713)	Class 4: low anxious-depression (*n* = 3,582)	*p*-values
Age, mean (SD)	73.13(8.069)	72.2(7.818)	71.59(7.617)	68.37(68.826)	0.000^a^
Sex, *n*(%)					0.055^b^
Male	162(42.7)	220(49.8)	364(51.1)	1778(49.6)	
Female	217(57.3)	222(50.2)	349(48.9)	1804(50.4)	
Total	379(7.4)	442(8.6)	713(13.9)	3,582(70.0)	
Living status, *n*(%)					0.015^b^
With household member(s)	333(87.9)	407(92.1)	653(91.6)	3,315(92.5)	
Living alone	46(12.1)	35(7.9)	60(8.4)	267(7.5)	
Total	379(7.4)	442(8.6)	713(13.9)	3,582(70.0)	
Yearly income, *n*(%)					0.123^b^
≤30,000 RMB	180(47.5)	209(47.3)	350(49.1)	1,600(44.7)	
>30,000 RMB	199(52.5)	233(52.7)	363(50.9)	1982(55.3)	
Total	379(7.4)	442(8.6)	713(13.9)	3,582(70.0)	
Educational level, *n*(%)					0.001^b^
No formal education or primary education	225(59.4)	250(56.6)	418(58.6)	1876(52.4)	
Secondary education	154(40.6)	192(43.4)	295(41.4)	1706(47.6)	
Total	379(7.4)	442(8.6)	713(13.9)	3,582(70.0)	
Social status, *n*(%)					0.004^b^
Social decline	74(19.5)	88(19.9)	127(17.8)	533(14.9)	
Active social interaction	305(80.5)	354(80.1)	586(82.2)	3,049(85.1)	
Total	379(7.4)	442(8.6)	713(13.9)	3,582(70.0)	
CSI-D total score, mean (SD)	4.99(4.009)	7.37(2.177)	8.10(1.826)	8.74(0.972)	<0.001^a^
GDS-15 total score, mean (SD)	8.34(2.601)	2.13(1.552)	3.41(1.146)	0.15(0.386)	0.000^a^
GAD-7 total score, mean (SD)	5.03(3.931)	2.15(2.037)	0.18(0.541)	0.02(0.150)	0.000^a^

^a^Chi-square test. ^b^Analysis of Variance (ANOVA).

### 3.3 The association between the anxious-depression and cognitive function

Significant variables from the simple linear regression analysis (Table 5) were incorporated into the multiple linear regression. The multiple linear regression analysis (Table 6) showed that age was negatively associated with cognitive function (Beta = −0.228, *p* < 0.001), whereas active social interaction was positively associated with cognitive function (Beta = 0.03, *p* = 0.011). Additionally, anxious-depression symptoms phenotypes remained significantly associated with cognitive function (Table 6). Compared with the low anxious-depression, the high anxious-depression, moderate anxious-depression, and high depression groups scored 0.467, 0.164, and 0.079 SD lower on the CSI-D, respectively (all *p* < 0.001). Although the CSI-D does not have an established minimal clinically important difference, the nearly half-standard-deviation reduction observed in the high anxious-depression group may indicate a meaningful difference in overall cognitive screening performance at the population level. In intergroup comparisons, compared to groups with a single symptom, those exhibiting both significant anxiety and depression symptoms demonstrated poorer cognitive function. Specifically, compared with the high depression, the moderate anxious-depression had significantly lower CSI-D scores by 0.1 SD (*p* < 0.001 for all comparisons), while the high anxious-depression had scores lower by 0.407 SD (*p* < 0.001 for all comparisons). Higher levels of anxious-depression were significantly associated with worse cognitive function. Compared with the moderate anxious-depression, the high anxious-depression exhibited cognitive scores 0.313 SD lower (*p* < 0.001 for all comparisons), representing a significant difference.

**Table 5:** Univariate linear regression analysis of cognitive function and anxious-depression.

Variables	Beta	SE	*p*-values
Age	−0.326	0.003	<0.001
Sex	−0.013	0.054	0.359
Living status	−0.065	0.1	<0.001
Yearly income	−0.02	0.054	0.152
Educational level	0.024	0.054	0.082
Social status	0.08	0.073	<0.001
High anxious-depression ^*^	−0.507	0.089	0.000
Moderate anxious-depression ^*^	−0.199	0.083	<0.001
High depression ^*^	−0.115	0.068	<0.001

*The low anxious-depression served as the reference group.

**Table 6:** Multivariate linear regression analysis of cognitive function and anxious-depression.

	Sample	Beta	SE	*p*-values *
Class 4	3,582	ref	ref	ref
Class 3	713	−0.079	0.066	<0.001
Class 2	442	−0.164	0.081	<0.001
Class 1	379	−0.467	0.087	<0.001
Class 2 vs. Class 3 (Ref)	NA	−0.1	0.096	<0.001
Class 1 vs. Class 3 (Ref)	NA	−0.407	0.101	<0.001
Class 1 vs. Class 2 (Ref)	NA	−0.313	0.111	<0.001

1. a) Class 1, high anxious-depression, b) Class 2, moderate anxious-depression, c) Class 3, high depression, d) Class 4, low anxious-depression; 2. Adjusting for covariates: age, living arrangements, and social status; 3. “*” indicates *P*-values adjusted using Bonferroni correction.

## 4 Discussion

This study aimed to assess emotional and cognitive function in community-dwelling older Chinese adults, and to examine the relationship between different anxious-depression phenotypes and cognitive decline. Using LCA, four distinct symptom profiles of anxious-depression were identified: high anxious-depression, moderate anxious-depression, high depression, and low anxious-depression. These phenotypes were found to effectively capture the demographic characteristics of the sample population. Elevated anxiety and depression symptoms were associated with lower cognitive function scores. Compared to older adults with minimal anxious-depression symptoms, those exhibiting prominent depressive symptoms alone or significant anxious-depression both exhibited significantly poorer cognitive function. Importantly, older adults with high levels of anxious-depression experienced the most pronounced cognitive decline, suggesting that coexisting anxiety and depressive symptoms may be associated with greater reductions in cognitive function in older adults. These findings highlight the potential impact of mental health issues on cognitive decline among older adults in community settings and underscore the importance of early identification and management of psychological symptoms to preserve cognitive health in aging populations.

In this community-based study, the prevalence rates of anxiety symptoms (4.4%) and depression symptoms (10.6%) observed in the study sample were significantly lower than those reported in a community-based study of older adults in Hunan Province (anxiety: 32.74%, depression: 37.34%) (29). However, it was comparable to the depression prevalence rate reported in Shenzhen (10.4%) and remains lower than the anxiety disorder detection rate reported in the region (11.3%) (3). Differences between studies may stem from variations in assessment tools, diagnostic thresholds, and sampling strategies employed. Moreover, they may reflect regional variations in economic development, healthcare access, demographic characteristics, and sociocultural factors.

This study identified four distinct symptom categories: high anxious-depression, moderate anxious-depression, high depression, and low anxious-depression. Among these, the symptom phenotypes of low, moderate, and high anxious-depression are consistent with those reported in studies from England (30), the United States (31), and Japan (19). In older adults with pronounced depressive symptoms (Classes 1–3), approximately half (Classes 1 and 2) also exhibited clinically significant anxiety symptoms. This finding aligns with previous epidemiological studies that have reported a relatively high comorbidity rate between depression and anxiety (5). Existing research evidence indicates that anxiety and depression co-occurrence share a common neurobiological basis (32, 33), providing a biological rationale for their frequent co-occurrence in both clinical and community populations. Notably, this study identified a “depression-dominant” subgroup characterized primarily by reduced interest/activity (D2), lack of spirits (D5), subjective memory complaints (D10), and lack of energy (D12). It should also be noted that the memory-related item in the GDS-15 primarily reflects subjective memory complaints rather than direct objective assessment of cognitive impairment (34). Therefore, the observed association between the depression-dominant subgroup and lower cognitive performance may partly reflect negative subjective perceptions of cognition commonly observed in depression. Nevertheless, previous studies have suggested that depressive symptoms may be associated not only with subjective cognitive complaints but also with genuine cognitive decline in older adults (35, 36).

This symptom pattern has not been observed in comparable studies of Western populations (5, 19), where an “anxiety-dominant” subgroup is more prevalent. The discrepancies in findings may be attributed to several factors. First, differences in the measurement tools employed may lead to variations in symptom classification results. Previous studies predominantly used the PHQ-9 or CES-D, whereas this study utilized the GDS-15. Research has shown that the GDS-15 tends to report a higher prevalence of depressive symptoms among Chinese older adults compared to other scales (37). Second, cultural factors may play a significant role in symptom expression (38, 39). Chinese patients tend to emphasize depressive mood and somatic symptoms of depression (40), while Western populations are more likely to report distress associated with anxiety (19, 41). Additionally, demographic characteristics and socioeconomic backgrounds may also exert an influence. Research indicates that among older adults, the prevalence of depression is higher than that of anxiety disorders compared to younger individuals (42). In developing regions, the prevalence of depressive symptoms is also higher than in high-income countries (43, 44). Common behavioral and psychosocial risk factors for late-life depression—including reduced physical activity (45), sleep disturbances (46), social withdrawal (47), and diminished motivation (31)—may further contribute to the heightened severity of depressive symptoms observed in this study. Therefore, the findings of this study reflect the diversity of anxiety and depression symptoms among older adults across different regions, while also underscoring the importance of employing person-centered latent class analysis methods to identify subtypes of anxiety and depression symptoms in community settings.

The findings of this study demonstrated a significant correlation between the anxious-depression phenotype and cognitive function scores. This result contrasted with the three anxious-depression groups (low, moderate, and high anxious-depression) identified in studies of Hispanic/Latino populations (48, 49). Notably, this study included an additional depression-dominant category through latent class analysis (LCA). The cognitive function analysis revealed that, compared to older adults with predominantly depressive symptoms, those with comorbid anxiety and depression exhibited more pronounced cognitive decline. This discrepancy may reflect differences in demographic characteristics, symptom presentation, and sociocultural backgrounds among the study populations. Previous research suggests that depression and anxiety may jointly affect cognitive function through shared neurobiological mechanisms, such as dysregulation of the stress response system and low-grade inflammation (50, 51). These pathways provide a biological explanation for the additive or synergistic effects observed in comorbid conditions. Beyond biological mechanisms, psychosocial and behavioral factors also play a significant role. Individuals experiencing concurrent anxiety and depression are more prone to sleep disturbances (46), social withdrawal (47), reduced physical activity (46), and diminished participation in cognitively stimulating activities (52), all of which may similarly accelerate cognitive decline. Therefore, our findings underscore the necessity of early identification and implementation of comprehensive mental health interventions among community-dwelling older adults to reduce their risk of developing cognitive impairment.

This study was the first to examine the association between anxious-depression symptom phenotypes and cognitive function in community-dwelling older adults in China, offering new insights into the interplay between emotional health and cognitive decline in the older adults. One of the key findings of this study was the identification of a symptom group primarily characterized by depression. In contrast to findings from western populations, these results may reflect how cultural, racial, and socioeconomic differences influence the expression and classification of anxiety and depressive symptoms. The study highlights the importance of early identification and comprehensive mental health interventions for older adults in community settings. However, several limitations should be acknowledged. First, the cross-sectional design prevents establishing a causal relationship between cognitive decline and anxiety/depression. Longitudinal follow-up studies are needed to clarify this association. Second, emotional and cognitive assessments were based on screening instruments rather than diagnostic tools. Although the CSI-D is a widely used and validated instrument for community-based cognitive assessment, it primarily evaluates global cognitive function and may not fully capture specific cognitive domains, particularly executive function and processing speed, that may be affected by anxiety and depressive symptoms. Future studies incorporating broader neuropsychological assessments are warranted to better characterize these relationships. In addition, a relatively large proportion of participants were excluded from the final analysis because of missing data. Comparisons between included and excluded participants suggested the possibility of selection bias. Accordingly, both internal validity and the generalizability of the findings should be interpreted cautiously. Finally, although all eligible older adults within the selected communities were invited to participate, the included communities were not randomly selected from all communities in Hefei, which may limit the generalizability of the findings to the broader older adult population.

In conclusion, four distinct anxious-depression symptom profiles were identified among community-dwelling older adults: high anxious-depression (7.41%), moderate anxious-depression (8.64%), high depression (13.94%), and low anxious-depression (70.01%). Cognitive function differed significantly across these profiles. Older adults with comorbid anxiety and depression exhibited more severe cognitive impairment than those with predominantly depressive symptoms. These findings highlight the importance of addressing comorbid anxiety and depression in preventing cognitive decline. Future research should focus on developing targeted interventions to reduce cognitive decline and improve health outcomes in older adults.

## Data Availability

The raw data supporting the conclusions of this article will be made available by the authors, without undue reservation and upon reasonable request.
