# Supplementary material for: Association between anxious-depression and cognitive function in community-dwelling older adults: a latent class analysis
Source: Front Public Health. 2026 Jun 26;14:1851644. doi: 10.3389/fpubh.2026.1851644 (PMC13352473; doi:10.3389/fpubh.2026.1851644)
Supplement: Supplementary file 1 [file Table_1.DOCX]

| **Variable** | **Included (n=5116)** | **Excluded (n=4293)** | **p-values** |
| --- | --- | --- | --- |
| Age，mean (SD) | 69.50(7.341) | 74.23(9.243) | <0.001^c^ |
| Sex, n (%) |  |  | 0.002^a^ |
| Male | 2524(49.3) | 1982(46.2) |  |
| Female | 2592(50.7) | 2311(53.8) |  |
| Educational level, n (%) |  |  | <0.001^a^ |
| No formal education or primary education | 2769(54.1) | 2786(64.9) | |
| Secondary education | 2347(45.9) | 1507(35.1) | |

**Table S1** Comparison of demographic characteristics between included and excluded participants.

Note: a, Chi-square test，c, Independent-samples t test.
